# Non-commutativity measure of quantum discord

**DOI:** 10.1038/srep25241

**Published:** 2016-04-28

**Authors:** Yu Guo

**Affiliations:** 1 School of Mathematics and Computer Science, Shanxi Datong University, Datong, Shanxi 037009, China

## Abstract

Quantum discord is a manifestation of quantum correlations due to non-commutativity rather than entanglement. Two measures of quantum discord by the amount of non-commutativity via the trace norm and the Hilbert-Schmidt norm respectively are proposed in this paper. These two measures can be calculated easily for any state with arbitrary dimension. It is shown by several examples that these measures can reflect the amount of the original quantum discord.

The characterization of quantum correlations in composite quantum states is of great importance in quantum information theory^1^,^2^,^3^,^4^,^5^,^6^. It has been shown that there are quantum correlations that may arise without entanglement, such as quantum discord (QD)^4^, measurement-induced nonlocality (MIN)^6^, quantum deficit^7^, quantum correlation induced by unbiased bases^8^,^9^ and quantum correlation derived from the distance between the reduced states^10^, etc. Among them, quantum discord has aroused great interest in the past decade^11^,^12^,^13^,^14^,^15^,^16^,^17^,^18^,^19^,^20^,^21^,^22^,^23^,^24^,^25^,^26^,^27^,^28^,^29^,^30^. It is more robust against the effects of decoherence^13^ and can be a resource in quantum computation^31^,^32^, quantum key distribution^33^ remote state preparation^34^,^35^ and quantum cryptography^36^.

Quantum discord is initially introduced by Ollivier and Zurek^4^ and by Henderson and Vedral^5^. The idea is to measure the discrepancy between two natural yet different quantum analogs of the classical mutual information. For a state *ρ* of a bipartite system A + B described by Hilbert space *H*_*a*_ ⊗ *H*_*b*_, the quantum discord of *ρ* (up to part B) is defined by


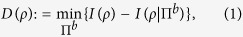


where, the minimum is taken over all local von Neumann measurements Π^*b*^, 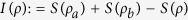 is interpreted as the quantum mutual information, 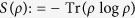 is the von Neumann entropy, 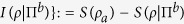, 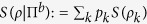 and 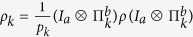 with 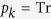 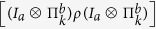, *k* = 1, 2, …, dim *H*_*b*_. Calculation of quantum discord given by Eq. (1) in general is NP-complete since it requires an optimization procedure over the set of all measurements on subsystem B^37^. Analytical expressions are known only for certain classes of states^15^,^16^,^20^,^38^,^39^,^40^,^41^,^42^,^43^,^44^,^45^. Consequently, different versions (or measures) of quantum discord have been proposed^19^,^24^,^25^,^46^,^47^: the discord-like quantities in^46^, the geometric measure^47^, the Bures distance measure^24^ and the trace norm geometric measure^19^, etc. Unfortunately, all of theses measures are difficult to compute since they also need the minimization or maximization scenario.

Let {|*i*_*a*_〉} be an orthonormal basis of *H*_*a*_. Then any state *ρ* acting on *H*_*a*_ ⊗ *H*_*b*_ can be represented by


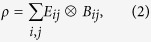


where *E*_*ij*_ = |*i*_*a*_〉〈*j*_*a*_| and 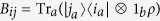. That is, assume that Alice and Bob share a state *ρ*, if Alice take an ‘operation’


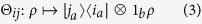


on her part, then Bob obtains the local operator *B*_*ij*_ (Note here that, the ‘operation’ Θ_*ij*_ is not the usual quantum operation which admits the Kraus sum respresentation). Quantum discord is from non-commutativity: *D*(*ρ*) = 0 if and only if *B*_*ij*_s are mutually commuting normal operators^47^,^48^. It follows that the non-commutativity of the local operators *B*_*ij*_s implies *ρ* contains quantum discord. The central aim of this article is to show that, for any given state written as in Eq. (2), its quantum discord can be measured by the amount of non-commutativity of the local operators, *B*_*ij*_s. In the following, we propose our approach: the non-commutativity measures. We present two measures: the trace norm measure and the Hilbert-Schmidt norm one. Both of them can be calculated for any state directly via the Lie product of the local operators. We then analyze our quantities for the Werner state, the isotropic state and the Bell-diagonal state in which the original quantum discord have been calculated. By comparing our quantities with the original one, we find that our quantities can quantify quantum discord roughly for these states.

## Results

### The amount of non-commutativity

Let *X* and *Y* be arbitrarily given operators on some Hilbert space. Then [*X*, *Y*] = *XY* − *YX* = 0 if and only if ||[*X*, *Y*]|| = 0, ||·|| is any norm defined on the operator space. That is, ||[*X*, *Y*]|| ≠ 0 implies the non-commutativity of *X* and *Y*. In general, ||[*X*, *Y*]|| reflects the amount of the non-commutativity of *X* and *Y*. Furthermore, for a set of operators Γ = {*A*_*i*_ : 1 ≤ *i* ≤ *n*}, the total non-commutativity of Γ can be defined by


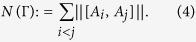


In ref. 49, *N*(Γ) is used for measure the ‘quantumness’ of a quantum ensemble Γ when ||·|| is the trace norm ||·||_Tr_, i.e., 
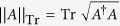
. We remark here that any norm can be used for quantifying the amount. It is a natural way that, for any state as in Eq. (2), the amount of its non-commutativity can be considered as the total non-commutativity of {*B*_*ij*_}, *N*({*B*_*ij*_}).

### Non-commutativity measure of quantum discord

Let 
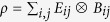
 be a state acting on *H*_*a*_ ⊗ *H*_*b*_ as in Eq. (2). We define a measure of QD for *ρ* by


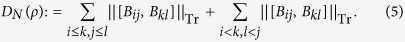


Similarly, we can define


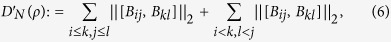


where ||·||_2_ denotes the Hilbert-Schmidt norm, i.e., 
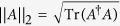
. That is, if Alice takes Θ_*ij*_s on her part, 1 ≤ *i*, *j* ≤ dim *H*_*a*_, then Bob can calculate the amount of non-commutativity through the reduced operators *B*_*ij*_s. By definition, it is obvious that i) *D*_*N*_(*ρ*) ≥ 0, 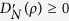, both *D*_*N*_ and 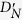 vanish only for the zero quantum discord states, i.e., 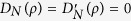 iff *D*(*ρ*) = 0; ii) both *D*_*N*_ and 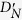 are invariant under the local unitary operations as that of the quantum discord, i.e., 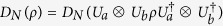 and 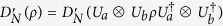 for any unitary operator *U*_*a*/*b*_ acting on *H*_*a*/*b*_ (this implies that *D*_*N*_ and 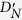 are independent on the choice of the local orthonormal bases: if 
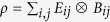
 with respect to the local orthonormal basis {|*i*_*a*_〉 |*j*_*b*_〉} and 
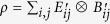
 with respect to another local orthonormal basis 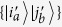, then 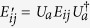 and 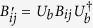 for some local unitary operators *U*_*a*_ and *U*_*b*_); iii) 
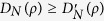
 for any *ρ*. By the definitions, it is clear that both *D*_*N*_ and 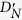 can be easily calculated for any state.

Let |*ψ*〉 be a pure state with Schmidt decomposition 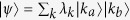. Then


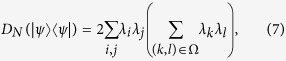



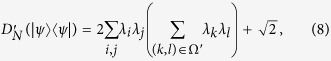


where Ω = {(*k*, *l*): either *i* < *k* ≤ *j* ≤ *l* or *k* = *i* and *l* = *j* if *i* < *j*; *i* ≤ *k* < *l* if *i* = *j*}, Ω′ = {(*k*, *l*): *i* < *k* ≤ *j* ≤ *l* if *i* < *j*; *i* ≤ *k* < *l* if *i* = *j*}. Therefore, *D*_*N*_(|*ψ*〉〈*ψ*|) = 0 (or 
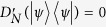
) if and only if |*ψ*〉 is separable. For the maximally entangled state 
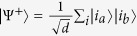
 in a *d* ⊗ *d* system, it is straightforward that 
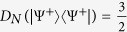
 whenever *d* = 2, 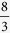 whenever *d* = 3 and 4 whenever *d* = 4, 
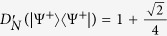
 whenever *d* = 2, 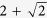 whenever *d* = 3 and 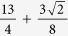 whenever *d* = 4. *D*_*N*_ and 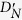 reach the maximum values only on the maximally entangled one.

It is worth mentioning here that both *D*_*N*_ and 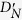 are defined without measurement, so the way we used is far different from the original quantum discord and other quantum correlations (note that all the measures of the quantum correlations proposed now are defined by some distance between the state and the post state after some measurement). In addition, it is clear that *D*_*N*_(*ρ*) and 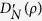 are continuous functions of *ρ* since both the trace norm and Hilbert-Schmidt norm are continuous. In^28^, a set of criteria for measures of correlations are introduced: (1) necessary conditions ((1-a)–(1-e)), (2) reasonable properties ((2-a)–(2-c)), and (3) debatable criteria ((3-a)–(3-d)). One can easily check that our quantity meets all the necessary conditions as a measure of quantum correlation proposed in^28^ (note that the condition (1-d) in^28^ is invalid for *D*_*N*_(*ρ*) and 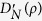). The continuity of *D*_*N*_ and 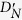 meets the reasonable property (2-a) (note: (2-b) and (2-c) are invalid since these two conditions are associated with measurement-induced correlation). (7) and (8) guarantee the debatable property (3-a). (3-c) and (3-d) are not satisfied as that of the original quantum discord while (3-b) is invalid for *D*_*N*_ and 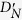. That is, all the associated conditions that satisfied by the original quantum discord are met by our quantities. From this perspective, *D*_*N*_ and 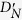 are well-defined measures as that of the original quantum discord.

### Comparing with the original quantum discord

**Figure 1:**
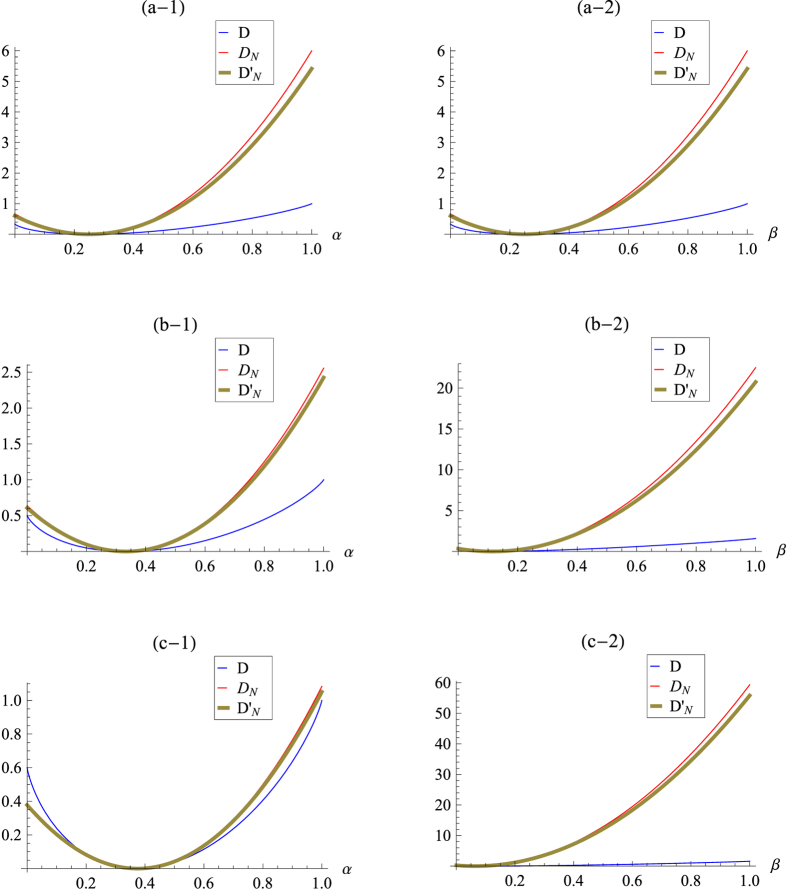
The measures *D*, *D*_*N*_ and 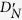 as a function of *α* for the Werner state when (**a-1**) *d* = 2, (**b-1**) *d* = 3 and (**c-1**) *d* = 4, and that of the isotropic state when (**a-2**) *d* = 2, (**b-2**) *d* = 3 and (**c-2**) *d* = 4. For both the Werner state and the isotropic state, *D*_*N*_ and 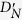 are monotonic functions of *D*.

**Figure 2:**
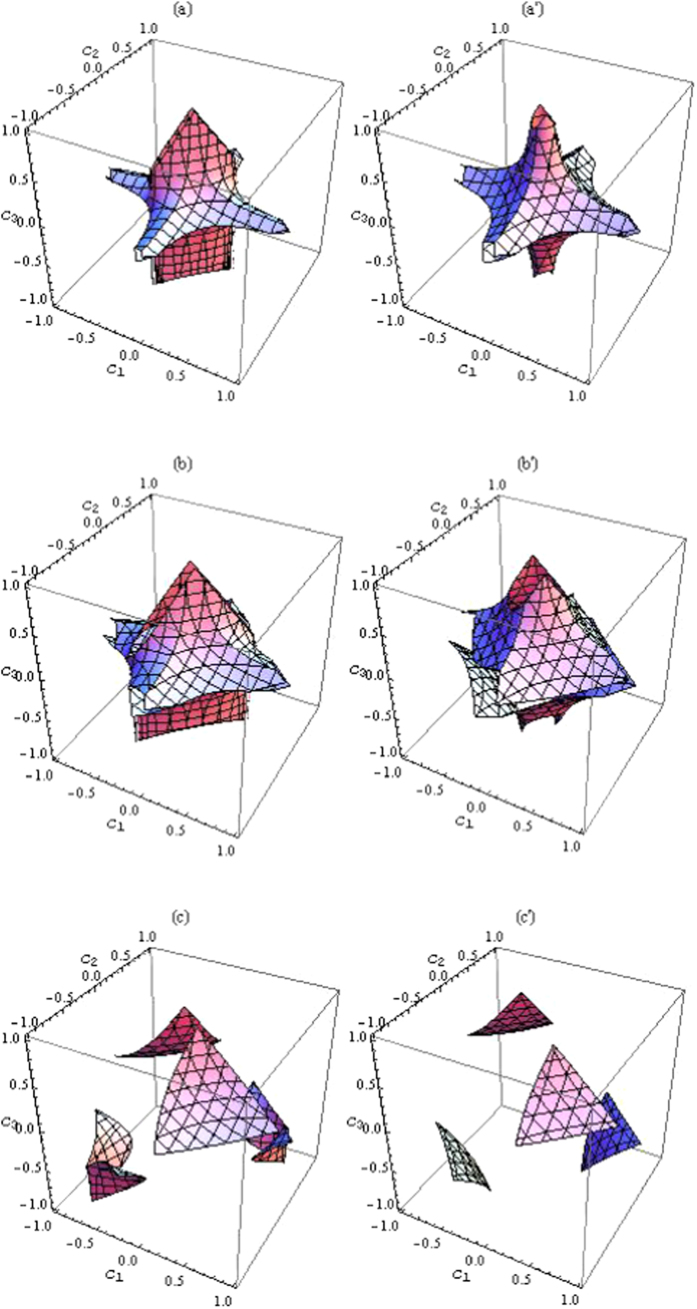
The surfaces of constant *D*_*N*_ and 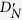 as a function of *c*_1_, *c*_2_ and *c*_3_ for: (**a**) *D*_*N*_ = 0.05, (**b**) *D*_*N*_ = 0.1 and (**c**) *D*_*N*_ = 0.3; (**a′**) 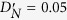, (**b′**) 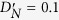 and (**c′**) 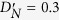.

**Figure 3:**
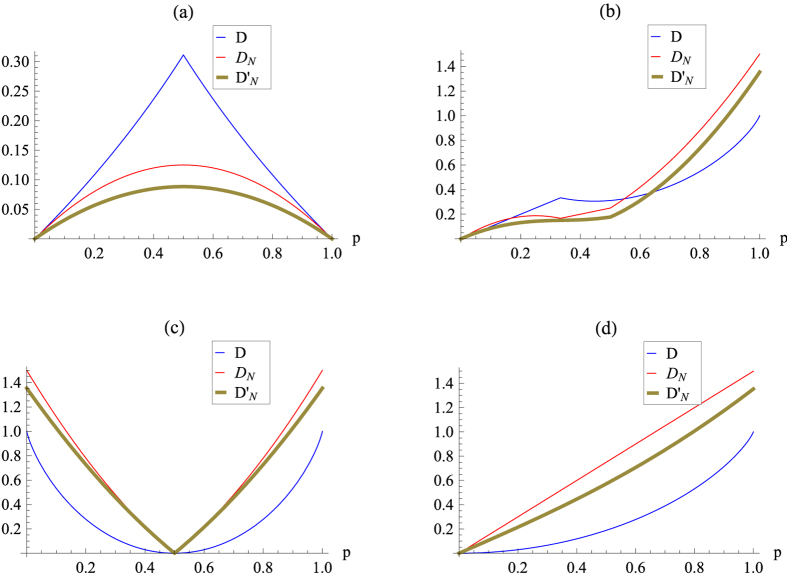
The measures *D*, *D*_*N*_ and 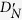 as a function of *p* for (**a**) *ρ*_1_, (**b**) *ρ*_2_, (**c**) *ρ*_3_ and (**d**) *ρ*_4_.

In what follows, we compare the non-commutativity measures *D*_*N*_ and 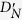 with quantum discord *D* for several classes of well-known states and plot the level surfaces for the Bell-diagonal states. These examples will show that *D*_*N*_ and 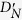 reflect the amount of quantum discord roughly: *D*_*N*_ and 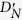 increase (resp. decrease) if and only if *D* increase (resp. decrease) for almost all these states (see Figs 1, 2, 3). *D*_*N*_ ≥ *D* and 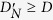 for almost all these states while there do exist states such that *D*_*N*_ < *D* and 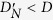 (see Fig. 3(a,b)). In addition, *D*_*N*_ and 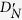 characterize quantum discord in a more large scale than that of *D* roughly. For the two-qubit pure state 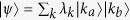, we can also calculate that 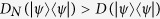 whenever *λ*_1_ > *a* with *a* ≈ 0.3841 while 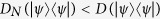 whenever *λ*_1_ < *a* and 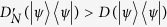 whenever *λ*_1_ > *b* with *b* ≈ 0.4279 while 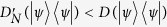 whenever *λ*_1_ < *b*.

### Werner states

The Werner states of a *d* ⊗ *d* dimensional system admit the form^50^,


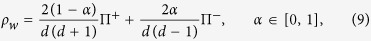


where 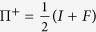 and 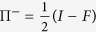 are projectors onto the symmetric and antisymmetric subspace of 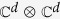 respectively, 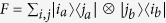 is the swap operator. Then


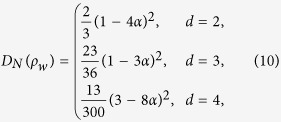


and


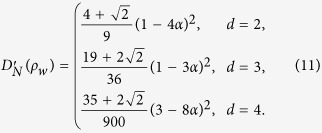


The three measures of quantum correlation, i.e., *D*_*N*_, 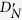 and *D*, are illustrated in (a-1), (b-1) and (c-1) in Fig. 1 for comparison, which reveals that the curves for *D*_*N*_ and 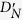 have the same tendencies as that of *D*.

### Isotropic states

For the *d* ⊗ *d* isotropic state


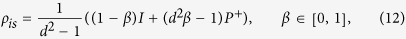


where 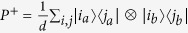 is the maximally entangled pure state in 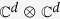. Then


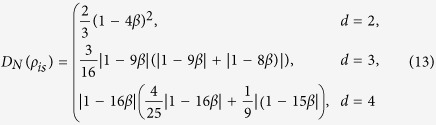


and


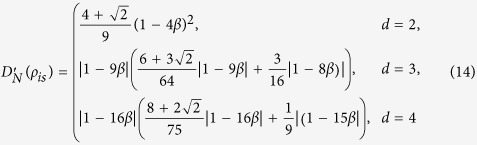


The three measures of quantum correlation, i.e., *D*_*N*_, 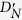 and *D*, are illustrated in (a-2), (b-2) and (c-2) in Fig. 1 for comparison. We see from this figure that the curves for *D*_*N*_ and 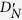 have the same tendencies as that of *D*. It also implies that i) for both the Werner states and the isotropic states, *D*_*N*_ and 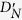 are close to each other, ii) *D* is close to *D*_*N*_ and 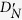 with increasing of the dimension *d* for the Werner states, which in contrast to that of the isotropic states.

### Bell-diagonal states

The Bell-diagonal states for two-qubits can be written as


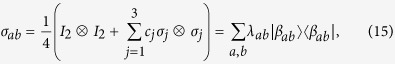


where the *σ*_*j*_s are Pauli operators, {|*β*_*ab*_〉} are four Bell states 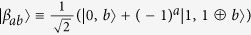. Then


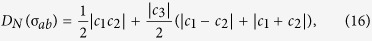



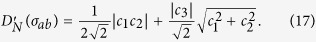


In Fig. 2, the level surfaces of *D*_*N*_ and 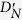 are plotted respectively. By comparing them with that of *D* in ref. 51, we find that the trends of *D*_*N*_ and 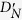 are roughly the same as that of *D*: *D*_*N*_ and 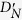 increase when *D* increases roughly and vice versa. (The geometry of the set of the Bell-diagonal states is a tetrahedron with the four Bell states sit at the four vertices, the extreme points of tetrahedron (i.e., (−1, 1, 1), (1, −1, 1), (1, 1, −1) and (−1, −1, −1)), see Fig. 1 in ref. 51 for detail.)

Especially, we consider


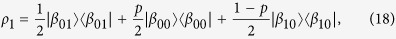



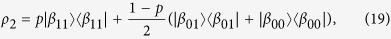



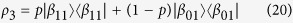


and


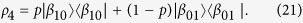


The three measures of quantum correlation, i.e., *D*_*N*_, 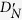 and *D*, are compared in Fig. 3. For *ρ*_1_, *ρ*_3_ and *ρ*_4_, the variation trends of *D*_*N*_ and 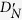 coincide with that of *D* while for *ρ*_2_ the curves of *D*_*N*_ and 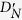 have the same tendency as that of *D* roughly. In addition, one can see that i) *D*_*N*_ and 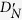 can both lager than and smaller than *D*, namely, there is no order relation between *D* and the two previous measures, ii) while the behavior of both measures *D*_*N*_ and 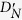 is quite similar, they are quite different from that of *D*.

Going further, we can quantify the symmetric quantum discord, i.e., the quantum discord up to both part A and part B. Let {|*k*_*b*_〉} be an orthonormal basis of *H*_*b*_, then any *ρ* acting on *H*_*a*_ ⊗ *H*_*b*_ admits the form


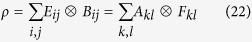


with *F*_*kl*_ = |*k*_*b*_〉〈*l*_*b*_|. Here, *A*_*kl*_ = Tr_*b*_(1_*a*_ ⊗ |*l*_*b*_〉〈*k*_*b*_|*ρ*) are local operators on *H*_*a*_. Let


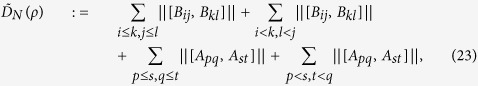


where ||·|| is the trace norm, or the Hilbert-Schmidt norm, or other norms. Then i) 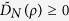 and 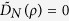 if and only if it is a classical-classical state (*ρ* is called a classical-classical state if 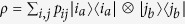 with *p*_*ij*_ ≥ 0 and 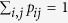); ii) 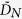 is invariant under the local unitary operations. We can conclude that 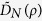 quantifies the amount of the symmetric quantum discord of *ρ*.

## Discussion

New measures of quantum discord has been proposed by means of the amount of the non-commutativity quantified by the trace norm and the Hilbert-Schmidt norm. Our method provides two calculable measures of quantum discord from a new perspective: unlike the original quantum discord and other quantum correlations were induced by some measurement, the two non-commutativity quantities we presented were not defined via measurements. Both of them can be calculated directly for any state, avoiding the previous optimization procedure in calculation. The nullities of our measures coincide with that of the original quantum discord and they are invariant under local unitary operation as well. The examples we analyzed indicate that, when comparing our quantities with the original quantum discord, although they are different and even have large difference for some special states, the non-commutativity measures reflect the original quantity roughly overall. We can conclude, to a certain extent, that our approach can reflect the original quantum discord for the set of states with arbitrary dimension. On the other hand, the non-commutativity measures reflect quantum discord in a larger scale than that of the original quantum discord, we thus can use these measures to find quantum states with limited quantum discord or the maximal discordant states (especially for the states represented by one or two parameters), etc.

As usual, only the trace norm and the Hilbert-Schmidt norm are considered. In fact we can also use the general operator norm or other norms in the definitions of *D*_*N*_ and 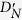. In addition, Fig. 2 shows that the level surfaces of 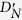 are nearly symmetric up to the four Bell states directions, which is very close to that of the quantum discord *D* (the level surfaces of *D* are symmetric up to the four Bell states directions^51^). Also note that the Hilbert-Schmidt norm is more easily calculated than the trace norm one, we thus use the Hilbert-Schmidt norm measure in general.

## Additional Information

**How to cite this article**: Guo, Y. Non-commutativity measure of quantum discord. *Sci. Rep.* **6**, 25241; doi: 10.1038/srep25241 (2016).
